# Mitigating Toxic Planktonic Cyanobacterial Blooms in Aquatic Ecosystems Facing Increasing Anthropogenic and Climatic Pressures

**DOI:** 10.3390/toxins10020076

**Published:** 2018-02-08

**Authors:** Hans W. Paerl

**Affiliations:** 1 Institute of Marine Sciences, University of North Carolina at Chapel Hill, Morehead City, NC 28557, USA; hpaerl@email.unc.edu; Tel.: +1-252-726-6841 (ext. 133); 2 College of Environment, Hohai University, Nanjing 210098, China

**Keywords:** cyanobacteria, nutrients (nitrogen and phosphorus), hydrology, climate change, water quality, bloom mitigation

## Abstract

Toxic planktonic cyanobacterial blooms are a pressing environmental and human health problem. Blooms are expanding globally and threatening sustainability of our aquatic resources. Anthropogenic nutrient enrichment and hydrological modifications, including water diversions and reservoir construction, are major drivers of bloom expansion. Climatic change, i.e., warming, more extreme rainfall events, and droughts, act synergistically with human drivers to exacerbate the problem. Bloom mitigation steps, which are the focus of this review, must consider these dynamic interactive factors in order to be successful in the short- and long-term. Furthermore, these steps must be applicable along the freshwater to marine continuum connecting streams, lakes, rivers, estuarine, and coastal waters. There is an array of physical, chemical, and biological approaches, including flushing, mixing, dredging, application of algaecides, precipitating phosphorus, and selective grazing, that may arrest and reduce bloom intensities in the short-term. However, to ensure long term, sustainable success, targeting reductions of both nitrogen and phosphorus inputs should accompany these approaches along the continuum. Lastly, these strategies should accommodate climatic variability and change, which will likely modulate and alter nutrient-bloom thresholds.

## 1. Introduction

**Figure 1:**
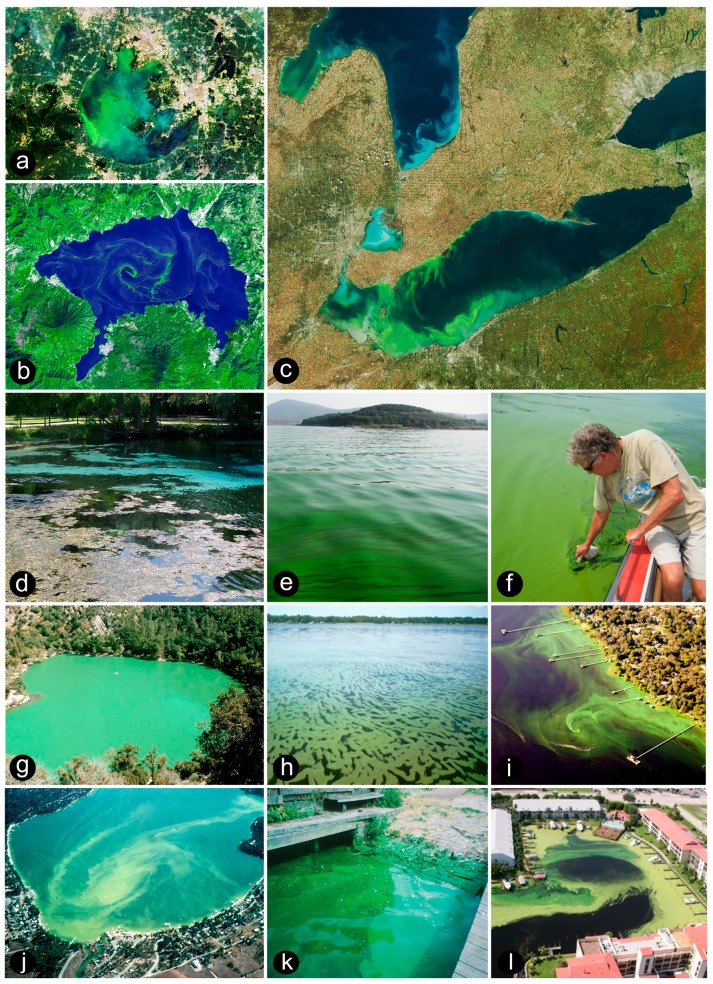
Cyanobacterial blooms, viewed for space and in the field. (**a**) MODIS satellite image of a summer (May 2007) *Microcystis* spp. bloom in lake Taihu, China (Courtesy NASA); (**b**) ASTER-TERRA image of a *Lyngbya* sp. Bloom in Lake Atitlan, Guatamala (Courtesy NASA); (**c**) MODIS image of *Microcystis*-dominated blooms in the Western Basin of Lake Erie and southern region of Saginaw Bay, Laurentian Great Lakes during the summer 2009 (Courtesy NASA and NOAA Coastwatch-Great Lakes); (**d**) Bloom of the benthic CyanoHAB *Lyngbya wollei* at Silver Glen Springs, Florida (Photo, Hans Paerl); (**e**) View of a *Microcystis*-dominated bloom in Meiliang Bay, Lake Taihu during summer 2009 (Photo, Hans Paerl); (**f**) Hans Paerl sampling the Taihu bloom during 2007; (**g**) Mixed *Microcystis* and *Dolichospermum* bloom in Zaca Lake, California, summer 1989 (Photo, Orlando Sarnelle); (**h**) Mixed *Microcystis*, *Anabaena*, and *Aphanizomenon* bloom in the St. Johns River, Florida, summer 1999 (Photo, John Burns); (**i**) Aircraft view of an *Anabaena* bloom on the St. Johns River (Photo, Courtesy of Bill Yates/CYPIX); (**j**) Mixed *Microcystis* and *Dolichospermum* bloom in Liberty Lake, Washington (Photo, Liberty Lake Sewer and Water District); (**k**) *Microcystis* bloom at launch ramp near Heemstede, The Netherlands, summer 1998 (Photo, Hans Paerl); (**l**) Mixed *Microcystis* and *Dolichospermum* bloom at a development near the Indian River Lagoon, Florida (Photo, John Burns).

**Figure 2:**
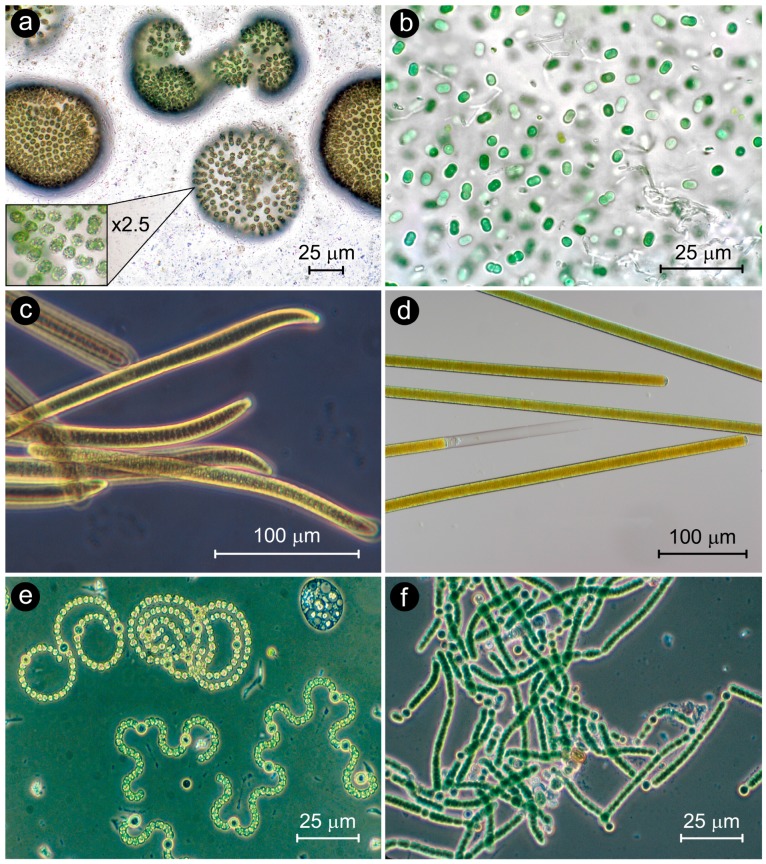
Photomicrographs of representative coccoid (**a**,**b**), filamentous non-heterocystous (**c**,**d**) and filamentous heterocystous (**e**,**f**) CyanoHAB genera. (**a**) *Microcystis* spp. (Photo, John Wehr); (**b**) *Synechococcus* sp. (Photo, Chris Carter); (**c**) *Oscillatoria* sp. (Photo, Hans Paerl); (**d**) *Lyngbya* sp. (Photo, Hans Paerl); (**e**) *Anabaena spiroides* (genus renamed *Dolichospermum*) and *A. circinalis* (Photo, Hans Paerl); (**f**) *Nodularia* sp. (Photo, Hans Paerl and Pia Moisander).

Cyanobacteria were the first oxygenic phototrophs that appeared on Earth, with fossil records indicating that they were present at least 2 billion years ago [1,2]. They were instrumental in forming an oxygen-rich atmosphere, one of the most significant biogeochemical changes to have impacted the biosphere. Today, their notoriety and evolutionary success continues, with some taxa dominating eutrophic waters as toxic, hypoxia-generating and food web altering blooms, or CyanoHABs (Figure 1 and Figure 2).

Harmful blooms occur in lakes, reservoirs, rivers, estuarine, and coastal waters [3] (Figure 1), where they can flourish in a wide variety of physically- and chemically-diverse as well as extreme environments. The ability of many cyanobacterial taxa to exploit these environments is likely related to their long evolutionary history in which they have “seen it all” with regard to major geochemical changes that the earth has experienced [2]; including periods of extreme cold, heat, desiccation, volcanism, and changes in solar radiation. Bloom-forming cyanobacterial genera are remarkably resistant to environmental extremes. They possess heat- and desiccation-tolerant resting cells (akinetes), sheaths and capsules, photoprotective pigments, and the ability to glide and rapidly move throughout the water column by altering their buoyancy, thereby optimizing access to light and nutrients [3,4]. Some genera can access the vast reservoir of atmospheric nitrogen via nitrogen (N_2_) fixation, which can circumvent nitrogen limitation [5], sequester iron by secreting siderophores [6], store phosphorus, nitrogen, and other essential nutrients [4], produce secondary metabolites, including compounds toxic to animals, including humans [7,8] (Table 1), and counter adverse and stressful environmental conditions, including photooxidation, elevated salinity, and related osmotic stress [7,8,9,10]. In addition, bloom-forming cyanobacteria participate in diverse consortia, and symbioses with a broad array of microorganisms, higher plants and animals, which help alleviate environmental stresses and limitations [11,12].

**Figure 3:**
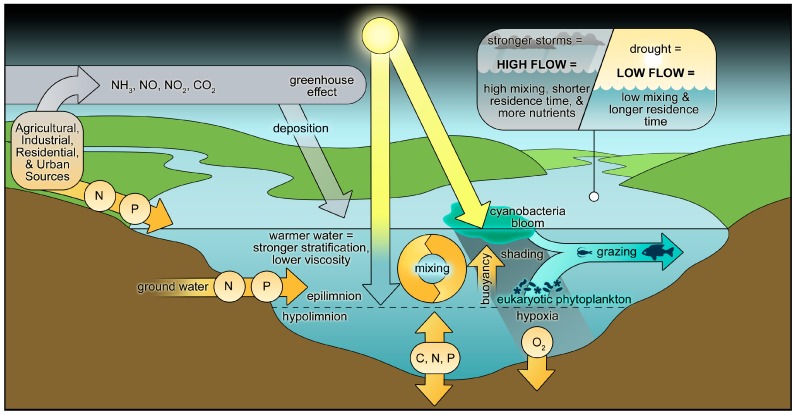
Conceptual diagram illustrating the various external and internal environmental and ecological factors controlling growth, accumulation (as blooms), and fate of CyanoHABs in freshwater ecosystems. Factors can act individually or in combined (synergistic, antagonistic) ways. They include: surface and subsurface as well as atmospheric nutrient inputs, physical controls, including mixing/circulation, freshwater inputs and flushing (i.e., residence time), light, temperature (including greenhouse gas mediated warming), grazing, and numerous within-system feedbacks, such as stratification and organic matter driven hypoxia, nutrient regeneration and light shading by blooms of subsurface phytoplankton populations. Lastly, physical forcing, such as wind-driven vertical mixing, can lead to sediment resuspension, which will impact light and nutrient availability.

These numerous intra- and extra-cellular adaptations are instrumental in countering and taking advantage of extreme conditions, due to either natural events or human alterations of the environment, including warming, changes in precipitation amounts and patterns, and subsequent hydrologic modifications, changes in flushing and water residence times and circulation, and nutrient over-enrichment (eutrophication) [8,13,14,15,16,17] (Figure 3). Cyanobacteria are well adapted to warming, with growth optima often exceeding 25 °C, in contrast to eukaryotic algal groups, which exhibit growth optima typically from 15–25 °C [14,15]. Furthermore, they exhibit metabolic flexibility, being able to optimize growth at either high or low nutrient (nitrogen, phosphorus, iron) supply rates. These adaptations are highly relevant in regimes of changing and extreme climatic conditions. They are also important to consider in the context of developing effective long-term mitigation strategies, as the adaptations discussed allow CyanoHABs to counter and take advantage of a range of environmental pressures.

Regionally and globally, significant climatic changes and extremes are taking place [18,19,20,21]. Climatic change and variability are known to profoundly affect the activities, distributions, and survivability of numerous plant and animal species [22], while benefiting CyanoHAB proliferation and dominance [14,15]. Therefore, the applicability and effectiveness of mitigation strategies are discussed in the context of a climatically and hydrologically more variable and extreme world.

## 2. CyanoHAB Mitigation Strategies

**Figure 4:**
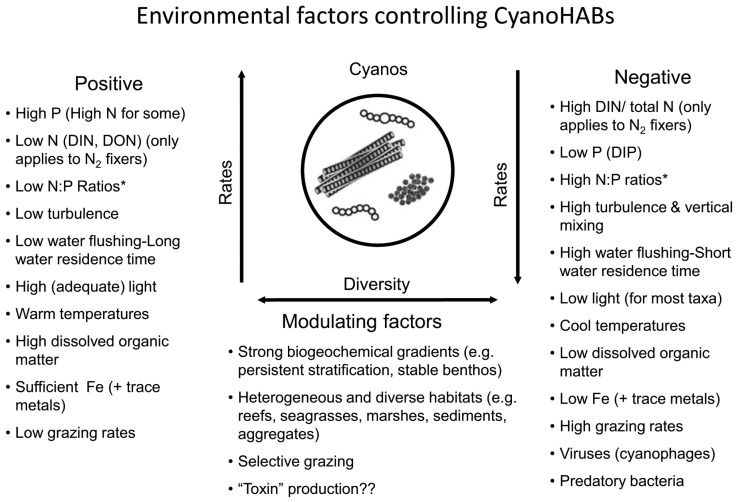
Summary of the positive and negative environmental and ecological effectors controlling CyanoHABs.

At the ecosystem scale, physical, chemical, and biotic regulatory variables may co-occur and interact synergistically and antagonistically to control key metabolic activities (photosynthesis, respiration, N_2_ fixation) and growth of CyanoHABs [7,13] (Figure 3 and Figure 4). Therefore, those controls that can effectively break this synergy are likely to be most effective and desirable from watershed and water quality management perspectives. These include (1) reducing nutrient inputs from external sources, (2) reducing internal release of nutrients already in the system, (3) increasing flushing rates of bloom-impacted waters, (4) destratifying such waters by artificial mixing or, (5) applications of algaecides, and (6) biological manipulations.

### 2.1. Nutrient-Based Mitigation Strategies

**Figure 5:**
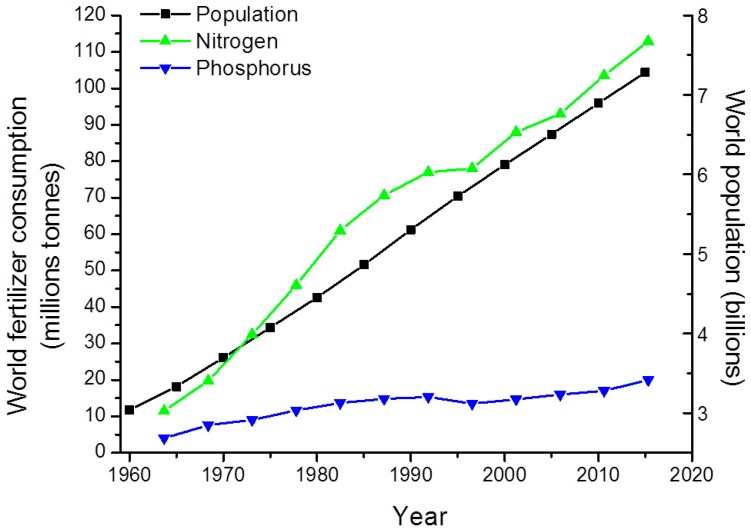
Global N and P-based fertilizer use in relation to the world’s human population. This underscores the tremendous increase in N fertilizer application, and resultant N losses to N-sensitive waters that has occurred in the past 50 years. This figure is adapted from FAO & United Nations data by Timothy Otten.

Excessive inputs of both phosphorus (P) and nitrogen (N) promote eutrophic and hypereutrophic conditions (waters having chlorophyll *a* concentrations often exceeding of 50 μg L^−1^ and regular bloom episodes accompanied by water quality/habitat impairment) [23,24,25]; a major result being chronic CyanoHABs [8,16,26,27]. The close connection between excessive nutrient loading and CyanoHAB frequencies and magnitudes forms a logical basis for prioritizing nutrient input constraints as a “bottom line” bloom mitigation strategy. The “nutrient problem” is especially acute in shallow lakes and reservoirs, where a legacy of P loading has led to P accumulation in sediments, and periodic sediment resuspension, as well as effective P regeneration form sediments ensures P availability. At the same time, global agricultural, urban, and industrial expansion has led to dramatic increases in N loading [28,29,30], while rates of P loading have stabilized or decreased, due in large part to the early recognition that P inputs play a key role in modulating freshwater eutrophication [31] (Figure 5).

Today, we are facing accelerating inputs from the growing use of synthetic N fertilizers, fossil fuel and agricultural atmospheric N emissions that are eventually deposited in watersheds or directly on the waterbody [32,33,34]. This has caused receiving waters to become relatively N-enriched, and in many instances more eutrophic [23,24,25,35]. Recent studies have shown that both freshwater and marine ecosystem can become even more eutrophic when they receive continued high rates of N loading. Interestingly, they continue exhibiting N limitation, despite increasing N loads [23,24,25,36]. Furthermore, there is mounting evidence that excessive N inputs tend to increase the production of N-containing cyanotoxins, especially microcystins [37].

N-enhanced eutrophication persists because: (1) some N is “lost” by denitrification. This leads to continuing demand for N supplies to sustain primary production and promote eutrophication [38,39], (2) a lengthy legacy of P loading that is stored in sediments, which is readily recycled and supports eutrophication, (3) N_2_ fixation, while providing “new” N, fails to meet ecosystem-scale N requirements [40,41]. From a nutrient management perspective, this means that continued external loading of N plays a critical role in accelerating eutrophication and promoting algal blooms [36].

**Figure 6:**
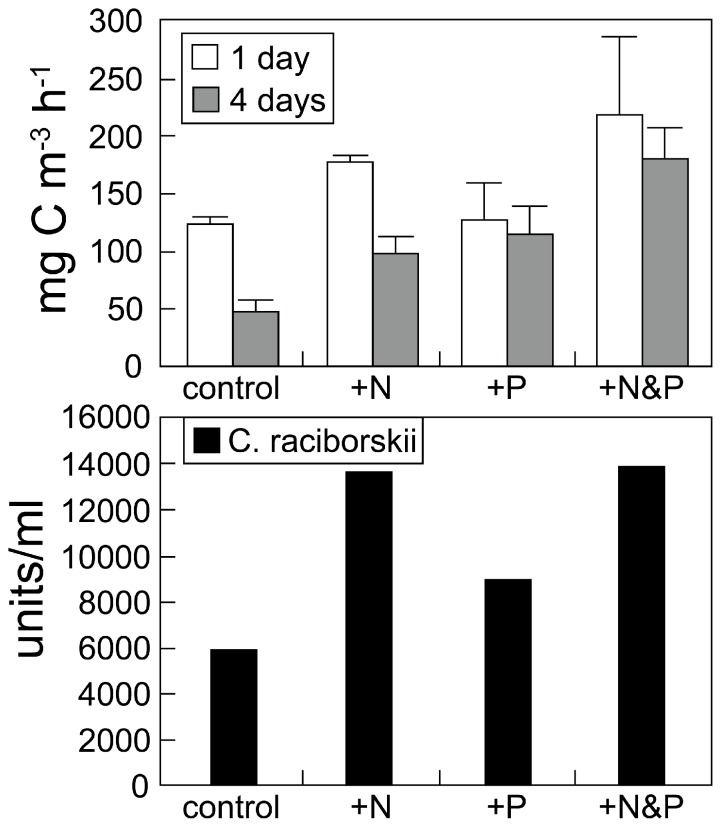
Results from a nutrient addition bioassay conducted on a naturally-occurring mixed cyanobacterial and eukaryotic algal bloom in the St. Johns River, Florida. Following nutrient additions, the bioassay was incubated under natural light and temperature conditions. **Upper graph:** effects of nitrogen (N, as nitrate), phosphorus (P, as phosphate) and combined nitrate and phosphate additions on rates of primary production (as CO_2_ fixation) after 24 and 48 h of incubation. **Lower graph:** effects of these nutrient additions specifically on the dominant CyanoHAB, the N_2_ fixer *Cylindrospermopsis raciborskii*, which was enumerated microscopically as numbers of filaments (units). Both the entire phytoplankton community and the *C. raciborskii* component revealed stimulation of primary production and growth in response to individual as well as combined N and P additions. From a nutrient management perspective, these results indicate that *both* N and P input reductions are needed to control the bloom.

One troubling indicator of N-driven eutrophication and CyanoHABs in freshwater systems is the geographic expansion and increase frequency of the non-N_2_ fixing toxin-producing genus *Microcystis* [42], especially in large lakes (e.g., Lake Erie, Canada-USA, Lake Kasumagura, Japan, Lake Okeechobee, Florida, USA, Lake Taihu, China). This genus, as well as another planktonic non-N_2_ fixing CyanoHAB *Planktothrix*, and several benthic analogue bloom taxa (e.g., non-N_2_ fixing *Lyngbya* and *Oscillatoria* spp.), serve as indicators of N-over-enrichment [36,42]. The proliferation of these non-N_2_ fixing CyanoHABs is an additional indicator the N input reductions are needed to reverse this trend. It has been pointed out that N_2_ fixing cyanobacterial species may replace non-N_2_ fixing ones in response to N reductions [43,44]. This scenario may be particularly troubling in lakes and reservoirs used for drinking water, fishing, and recreational purposes, because some toxic non-N_2_ fixing genera (e.g., *Microcystis*) may be replaced by toxic N_2_ fixers (e.g., *Aphanizomenon* spp., *Cylindrospermopsis raciborskii*, *Dolichospermum* spp., *Nodularia* spp.). This potential scenario should be carefully monitored for in waterbodies in which N-input reductions are being considered to reverse eutrophication, and control CyanoHABs. Work on hypereutrophic lakes like Lake Taihu, China, has shown that this is not the case, most likely because this system has experienced excessive loading and a legacy of internal storage of P and N that has existed for several decades [36,45]. In situ nutrient addition bioassays on such CyanoHAB-infested waters demonstrate the need for dual nutrient reduction strategies [27,36]. An example is shown for the St. Johns River, Florida, which supports blooms of both N_2_ and non-N_2_ fixing CyanoHABs (e.g., *Dolichospermum*, *Aphanizomenon*, *Microcystis*). Here, the addition of N and P led to greater stimulation of primary production and biomass than N or P alone (Figure 6).

A key management priority is establishing the extent of N and P reductions needed (i.e. nutrient input thresholds and total maximum daily loads, or TMDLs), effective at controlling CyanoHABs [45]. N to P input ratios should also be considered when developing such thresholds, and how they affect CyanoHAB toxicity [46]. Recent analyses have indicated that there is no “magic bullet” with regard to an ideal N/P input ratio that can consistently control both CyanoHAB species and their toxicity [46].

In addition to using N/P ratios, predicting CyanoHAB development, magnitude, and persistence, as well as toxicity, is reliant on a suite of interacting waterbody geological and hydrological properties. These include depth, area, and volume, the depth of mixing, and water residence or replacement time [17]. Most reliably, both total nutrient loads and concentrations must be considered in conjunction with these properties on a system-level basis for CyanoHAB management [9]. While it is often stated that molar N/P ratios above ~15 discourage CyanoHAB dominance [44,47], the ratio approach is neither effective nor meaningful if the nutrient load and internal concentrations of either N or P exceed the nutrient uptake saturation levels for specific CyanoHABs [46].

On the ecosystem scale, nutrient input sources are classified as point or non-point sources [48,49]. Point sources are identifiable discharge sites such as “pipelines” or conduits originating from industrial sites, wastewater treatment plants, and specific discharge points. In urban and industrial regions, point sources can account for a significant portion of nutrient loads, and they are generally accessible, and thus, relatively attractive and amenable for control. By contrast, diffuse, non-point sources, while frequently constituting a significant fraction of total loads, are often far more difficult to control [48,49,50].

Globally, non-point surface and subsurface nutrient sources are of increasing concern. This is due to growing use of chemical fertilizers, increased discharge of waste from confined animal operations, land conversion of forests and grasslands for agricultural use, increasing numbers of septic waste systems, groundwater contamination, and atmospheric deposition [48,49,50]. Non-point sources now account for over 50% of annual P loading alone in agricultural watersheds [48,49,50]. Because they frequently constitute a large fraction of total loads, they must be an integral component of comprehensive basin-wide nutrient reduction strategies. Relatively “low technology” but effective best management approaches are now available, and should be encouraged in agriculturally-dominated watersheds. These include the implementation of riparian vegetative buffers, constructed wetlands, and when feasible, no-till practices [49,50,51].

In natural waters, phosphorus is available as dissolved ionic, dissolved organic, and particulate forms. The dominant dissolved inorganic P (DIP) form is orthophosphate (PO_4_^3^^−^), which is generally available to CyanoHABs. Cyanobacteria are able to accumulate and intracellularly store assimilated DIP as polyphosphates, which serve as available P sources when external P sources are depleted [52]. Dissolved organic N (DOP) can be assimilated by bacteria, microalgae, and most cyanobacteria, but not as rapidly as PO_4_^3^^−^ [53]. Assimilated P can be rapidly recycled through microbial decomposition. Particulate P (PP) can be a sorption/precipitation site for DIP and DOP, with the possibility of subsequent desorption. These multiple P uptake and exchange pathways ensure effective recycling, and ensure that PP exists in dynamic equilibrium with the dissolved, biologically-available forms of P. The sediments constitute an often large and important “legacy” of stored P, which can be available for subsequent release, especially under low oxygen conditions (hypoxia, anoxia). Clearly, both dissolved and particulate P sources must be considered when managing P inputs and inventories.

Nitrogen is present in dissolved, particulate, and gaseous forms, and most forms are biologically available and effectively cycled in the water column and sediments [54]. Microbial (N_2_) fixation and denitrification mediate the air–water and sediment–water exchange between inert gaseous atmospheric N_2_ and biologically-available combined N species, including ammonia/ammonium (NH_3_/NH_4_^+^), nitrate (NO_3_^−^), and nitrite (NO_2_^−^). Additional biologically-available forms of N include dissolved organic N (DON; e.g., amino acids and peptides, urea, organo-nitrates), and particulate organic N (PON; polypeptides, proteins). These forms can be supplied from non-point and point sources, and many soluble forms are biologically available to CyanoHABs.

N inputs are closely linked to human activities, including land use, population density, and economic activity [30,33,48]. As such, the inputs and routes of N loading are highly dynamic [48]. The dominant sources of human N loading are surface runoff, groundwater, and atmospheric deposition [48].

### 2.2. Altering Sediment Nutrient Dynamics

A waterbody’s sediments represent the cumulative site and legacy of nutrient loading. In essence, the sediments act as the storage bank of nutrients that can be exchanged with and rapidly cycled between the bottom and water column. Sediments also act as an inoculum for CyanoHAB resting stages or spores, and in this manner, act as a “seed bank”. This knowledge has been used as a rationale for either removing or capping sediments as a mitigation strategy.

While dredging sounds attractive from a nutrient removal perspective, it is expensive, alters biogeochemical cycling in often unknown ways, disturbs the benthic floral and faunal habitat, and can lead to release of toxic substances that have accumulated there. Despite these potential drawbacks, there are some “success stories” involving dredging to remove excess nutrients that have reversed eutrophication and led to a decrease in CyanoHABs. One example is Lake Trummen, Sweden, a small (~1 km^2^), shallow (mean depth 1.6 m) lake that suffered from CyanoHAB outbreaks as a result of domestic sewage and industrial nutrient inputs during the mid-1900s [55]. Suction dredging the upper half meter of sediments for two years led to significant decreases in nutrient concentrations and CyanoHABs [56]. Dredging proved effective in Lake Trummen because it is small, and also has a small watershed (~13 km^2^) which was targeted for parallel reductions of nutrient inputs [57]. Sediment dredging in large lakes has been far less successful, or not noticeable at all, with regard to improving water quality and CyanoHAB outbreaks (e.g., Lake Taihu, China).

A less physically-disruptive approach is to use chemical treatment of lakes to precipitate P, keeping it “locked up” in the sediments. Traditionally, treatment of water bodies with potassium aluminum sulfate (alum) or iron salts has been used to precipitate phosphorus [55]. While such treatments can be effective in immobilizing P in highly managed ponds, it is difficult to maintain P in an immobilized state in vertically stratified systems where hypoxic and anoxic bottom waters can cause chemical release and subsequent availability of P. Furthermore, alum can be toxic to aquatic biota, and additions of iron can lead to unwanted consequences, including potentially stimulating algal blooms in aquatic systems which may exhibit iron limitation. An alternative treatment, called “Phoslock”, employs a bentonite clay containing lanthanum [58]. The lanthanum ions, which are bound to the bentonite, also strongly bind phosphate, which is then precipitated and settled on the sediment surface, where it forms a diffusive barrier, hence “locking” phosphorus in the sediments [58]. Phoslock has shown promise in small lakes, especially those that undergo summer stratification (i.e., minimizing resuspension), thus promoting P-limited conditions that help minimize CyanoHAB outbreaks [58,59]. The Phoslock layer also increases the critical erosional velocity of surficial sediments, thus minimizing resuspension events and potential release of P through pulse nutrient loading. Sediment stabilization and reduced phytoplankton biomass may also help with restoration of macrophyte communities in shallow, eutrophic water bodies, especially if planktonic production decreases and alleviates light limitation.

### 2.3. Hydrologic Manipulations

Hydrologic modifications can also be used to mitigate CyanoHABs (Figure 3 and Figure 4). Artificial mixing, either by air bubbling or mechanical mixing, can reduce water column stratification and enhance vertical mixing of the phytoplankton, thereby minimizing buoyant surface cyanobacterial blooms [8,60]. Enhancing horizontal flushing reduces water residence time, limiting the time for development of CyanoHABs [61,62]. While this approach can lead to suppression of CyanoHABs, especially in small water bodies, it is expensive, and requires a large, low-nutrient freshwater supply, which may be limited, or compete with other uses of such water supplies (drinking water, irrigation).

### 2.4. Application of Algaecides and Biomanipulation

The application of algaecides, most commonly copper-containing compounds [63], and more recently, hydrogen peroxide [64], have largely focused on relatively small impoundments, for obvious reasons, which include scale and expense. These mitigation steps require intensive and repetitive application, the spatial extent (i.e. radius of effectiveness) of their application can be quite limited, and they can be quite expensive to apply, deploy, and maintain. While copper-containing compounds can be effective algaecides, they are toxic to various aquatic plants and animals, and their residue in sediments constitutes a legacy pollutant [63]. Hydrogen peroxide (H_2_O_2_) is selective for cyanobacteria (vs eukaryotic algae and higher plants), and has minimal adverse effects, because it is broken down to H_2_O [64]. The H_2_O_2_ treatment must be repeated, however, especially if the period for bloom potentials is several months, because it is rapidly degraded rapidly to H_2_O.

The application of algaecides must be treated with caution, because upon death and lysis of the toxin-producing CyanoHABs, toxins will be released into the ambient waters. This can lead to contamination of drinking and irrigation water, and could bar recreational use of impacted waters. One additional benefit of H_2_O_2_ treatment is that light-stimulated oxidation by peroxide can break down microcystins, thus simultaneously detoxifying CyanoHAB-impacted waters [64].

Biomanipulation has also been attempted as a CyanoHAB mitigation strategy. This usually involves introduction of fish or benthic filter feeders as consumers of cyanobacteria, or the introduction of lytic bacteria and viruses. Key elements of biomanipulation are to both reduce CyanoHAB biomass and recycling of nutrients. A common strategy is to increase the abundance of herbivorous zooplankton, the most immediate consumers of phytoplankton biomass. This is usually accomplished by removing zooplanktivorous fish and/or introducing predatory piscivorous fish. In addition, benthivorous fish can also be removed, which provides the added benefit of reducing sediment resuspension and introduction of nutrients into the water column. There is a great deal of system-specific individuality with regard to taking these steps, because grazing pressure, internal nutrient cycling, and specific targeting of CyanoHABs can vary significantly on spatial and temporal scales. Care must also be taken to avoid selective dominance by toxin-producing CyanoHAB species, which may be avoided by grazers [65,66]. Because of its experimental nature, and at times, unpredictable outcomes, biomanipulation should probably only be considered when nutrient reductions alone are not effective in restoring acceptable water quality [67,68,69].

### 2.5. Climate Change and CyanoHAB Potentials

**Figure 7:**
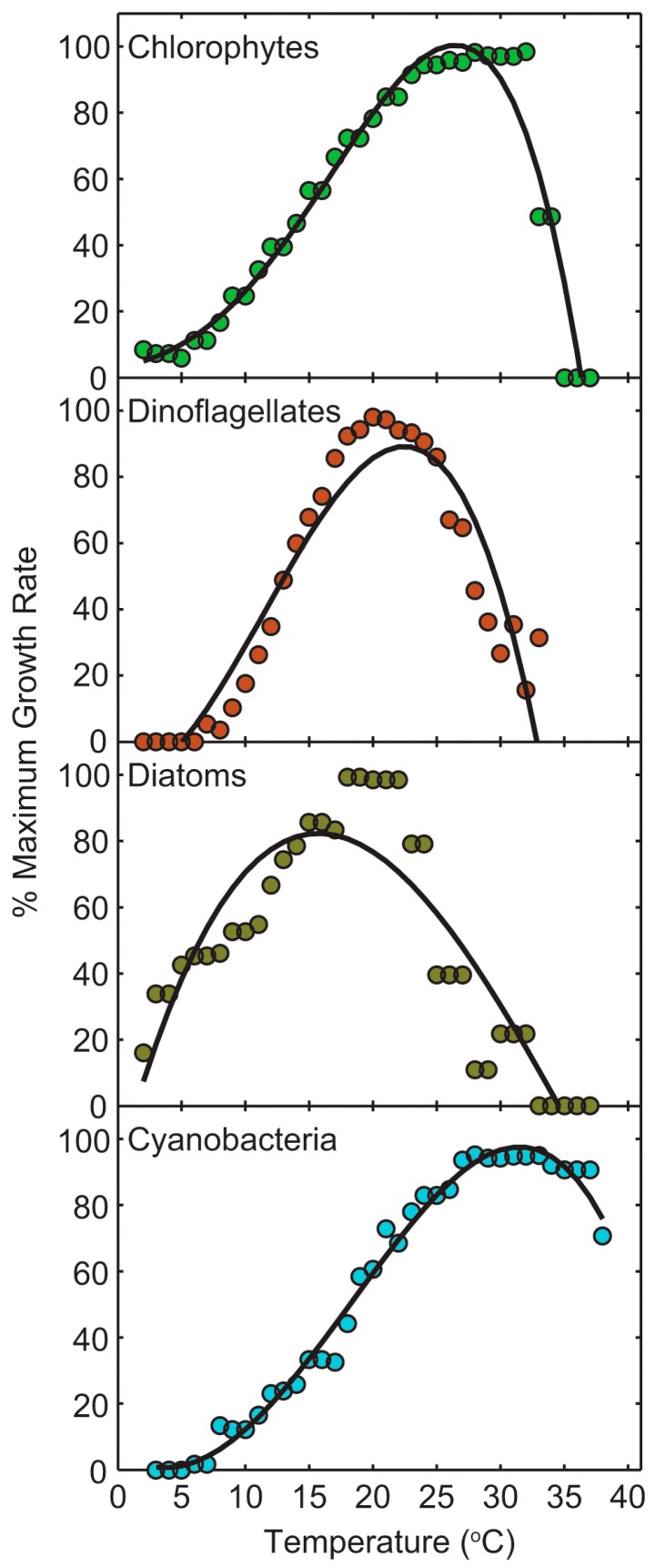
Effect of temperature on growth rates of major phytoplankton groups and CyanoHAB species common to temperate freshwater and brackish environments. Data points are 5 °C running bin averages of percent maximum growth rates from 3–4 species within each class. Fitted lines are third order polynomials, and are included to emphasize the shape of the growth versus temperature relationship. Percent maximum growth rates of individual species are provided in [16], reproduced with permission from [9]. Copyright Springer, 2013.

Climate change, specifically global warming and altered precipitation patterns and amounts, interact synergistically with nutrient enrichment to promote CyanoHABs [14,15,70,71,72]. Warming favors cyanobacteria, including CyanoHABs, because they show a strong preference, growth rate-wise, for relatively high temperatures, often exceeding 25 °C [73,74,75] (Figure 7). By contrast, eukaryotic algae exhibit growth optima at temperatures below 25 degrees [76,77,78,79,80,81,82] (Figure 7), giving cyanobacteria an advantage as surface waters warm, and warming begins earlier and lasts longer during the growth season, typically ranging from mid-spring to autumn [79,80,81,82].

Warmer surface waters will lead to intensification of vertical stratification, due to stronger density differences between the warm upper mixed layer and the colder hypolimnion. In marine systems, salinity gradients additionally strengthen stratification. [79,80,81,82] (Figure 3 and Figure 4). In lakes, reservoirs and even estuarine waters that are periodically ice covered (e.g., Baltic Sea), a warmer climate will cause the ice cover to melt earlier and refreeze later, leading to earlier and more extensive vertical stratification and a longer period of illumination of surface waters, thus extending the potential bloom period. This scenario has already been observed in lakes in northern Europe and North America; some of them are no longer ice-covered at all [76,79,80]. From a nutrient mitigation perspective, this means that point source N and P input controls will likely need to occur earlier and extended later in the year, while non-point nutrient (agricultural and urban stormwater runoff) controls will need to be carried out on a year-round basis in most watersheds.

Greater variability and more extremeness in the amounts of precipitation are also occurring with climate change. Major storms, including tropical cyclones, nor’easters, and summer thunderstorms, have exhibited higher amounts and intensities of rainfall [18,83], while droughts are more severe and protracted [18]. These greater oscillations in precipitation have led to greater hydrologic variability, i.e., wetter wet periods and drier dry periods. Greater episodic discharge events bring with them large freshets with pulse loads of nutrients to downstream waters. When followed by periods of extended drought, during which flow decreases and residence time increases, conditions are favorable for cyanobacterial blooms. If this scenario is accompanied by rising temperatures, “perfect storm” synergism exists for optimal bloom development and persistence [73,75].

Mitigating the “perfect storm” scenario calls for more vigilance with regard to timely fertilizer applications at agronomic rates, i.e., rates that optimize terrestrial plant growth, but avoid going beyond this. It also calls for increased development of riparian vegetative buffers in regions susceptible to storms, rapid runoff, and flooding events. These buffers, along with constructed wetlands and stormwater retention ponds, can be highly effective in processing and retaining soluble, as well as sediment-associated forms of N and P [49,51].

Higher amounts of freshwater runoff can enhance vertical density stratification in estuarine and coastal waters, as well as saline lakes, where relatively light freshwater lenses can form over denser saltwater. This will favor CyanoHABs capable of vertical migration to position themselves at physically and chemically optimal depths [84]. Buoyancy-regulating CyanoHABs can also orient themselves along light, temperature, and nutrient regimes, and to escape grazers which often avoid surface waters [4,8]. Most surface dwelling CyanoHABs possess photoprotective pigments, enabling them to counter excessive irradiance [85], while shading sub-surface phytoplankton, leaving them at sub-optimal light conditions (Figure 3). Enhancing destratification, using vertical mixing devices, can be effective in countering the buildup of surface CyanoHABs, especially in relatively small impoundments, and artificial water bodies, such as reservoirs and fish ponds [60]; however, for long-term CyanoHAB control, parallel nutrient input controls should be implemented.

## 3. Conclusions

Toxic CyanoHABs are rapidly expanding on the global scale, promoted by the synergistic interplay of excessive anthropogenic nutrient loading and increasingly favorable climatic conditions, including warming and increasing hydrologic variability. The long evolutionary history of cyanobacterial bloom taxa has led to both tolerance and adaptability to man-made and acute and longer term geological and climatically-induced environmental change. The most notable and controllable human alterations include: (1) nutrient (especially N and P) enrichment, (2) hydrological modifications, including reservoir construction, water use and diversions for consumption, irrigation, and flood control, (3) biological alterations, including negative impacts of aquaculture, overfishing, and introduction of exotic species, which can have profound trophodynamic impacts, (4) the use and introduction of toxins and xenobiotic compounds, e.g., heavy metals, herbicides and pesticides, industrial and domestic chemicals, antibiotics, and other synthetic growth regulators, which can affect phytoplankton community structure and function.

Long term CyanoHAB mitigation should focus on breaking the synergy between nutrient enrichment and physical–chemical impacts of climate change. This involves manipulating the broad suite of environmental factors known to promote CyanoHABs, along with knowledge of the ecological and physiological adaptations that certain taxa possess to circumvent specific controls, including, for example, the ability of N_2_ fixing taxa to exploit N-limited conditions, and the ability of buoyant CyanoHAB taxa to counter artificial mixing. Lastly, we must better understand the ecophysiological roles that the suite of secondary metabolites, including those toxic to consumers, play in the physiology and ecology of bloom-forming cyanobacteria. The synthesis of focused laboratory experimental work with ecosystem-level studies will prove invaluable in unraveling the complexity of environmental regulation and mitigation of CyanoHABs.

Ecosystem scale mitigation strategies will have to incorporate nutrient (focusing on nitrogen and phosphorus) input reductions; the magnitude and spatio-temporal extent of which extent are system-specific, and likely requiring adjustment with changing climatic conditions. A key long-term control we can exert to reduce the rate and extent of global warming is curbing greenhouse gas emissions. Without this essential step, future warming trends and their impacts on aquatic ecosystems will play into the hands of the opportunistic and rapidly-expanding CyanoHABs.

## Figures and Tables

**Table 1:** Various bloom-forming cyanobacterial genera, potential toxins they produce, morphological characteristics, preferred habitats, and salinity ranges they occupy. Table courtesy of T. Otten and adapted from [9].

Genus	Potential Toxin(s)	Characteristic	Salinity Range		
			Low (0–4)	Mod. (4–16)	High (16+)
*Anabaenopsis*	MC	P,D,F	X	X	X
*Aphanizomenon*	ATX, CYN, STX	P,D,F	X	X	
*Cylindrospermopsis*	ATX, CYN, STX	P,D,F	X		
*Cylindrospermum*	ATX, MC	B,D,F	X		
*Dolichospermum*	ATX, CYN, MC, STX	P,D,F	X	X	
*Fischerella*	MC	B,D,F	X	X	X
*Hapalosiphon*	MC	B,D,F	X		
*Lyngbya*	CYN, LYN, STX	B,F	X	X	X
*Microcystis*	MC	P,C	X		
*Nodularia*	NOD	B/P,D,F	X	X	X
*Nostoc*	ATX, MC	B,D,F	X	X	
*Oscillatoria*	ATX, CYN, MC, STX	B/P,D,F	X	X	X
*Phormidium*	ATX, MC	B,F	X	X	X
*Planktothrix*	ATX, MC	P,F	X	X	
*Raphidiopsis*	ATX, CYN, MC	P,F	X	X	
*Scytonema*	MC, STX	B,D,F	X	X	X
*Umezakia*	CYN, MC	P,D,F	X		

**Toxin abbreviations**: ATX = Anatoxin-a; BRV = Brevetoxin; CYN = Cylindrospermopsin; DA = Domoic acid; ICX = Ichthyotoxins; LYN = Lyngbyatoxin; MC = Microcystin; NOD = Nodularin; STX = Saxitoxin; **Characteristics abbreviations**: B = Benthic; C = Coccoid; D = Diazotrophic; F = Filamentous; P = Planktonic.
